# Spatial Heterogeneity of SOM Concentrations Associated with White-rot Versus Brown-rot Wood Decay

**DOI:** 10.1038/s41598-017-14181-7

**Published:** 2017-10-23

**Authors:** Zhen Bai, Qiang Ma, Yucheng Dai, Haisheng Yuan, Ji Ye, Wantai Yu

**Affiliations:** Institute of Applied Ecology, Chinese Academy of Sciences, Shenyang, 110016 China

## Abstract

White- and brown-rot fungal decay via distinct pathways imparts characteristic molecular imprints on decomposing wood. However, the effect that a specific wood-rotting type of fungus has on proximal soil organic matter (SOM) accumulation remains unexplored. We investigated the potential influence of white- and brown-rot fungi-decayed *Abies nephrolepis* logs on forest SOM stocks (i.e., soil total carbon (C) and nitrogen (N)) and the concentrations of amino sugars (microbial necromass) at different depths and horizontal distances from decaying woody debris. The brown-rot fungal wood decay resulted in higher concentrations of soil C and N and a greater increase in microbial necromass (i.e., 1.3- to 1.7-fold greater) than the white-rot fungal wood decay. The white-rot sets were accompanied by significant differences in the proportions of the bacterial residue index (muramic acid%) with soil depth; however, the brown-rot-associated soils showed complementary shifts, primarily in fungal necromass, across horizontal distances. Soil C and N concentrations were significantly correlated with fungal rather than bacterial necromass in the brown-rot systems. Our findings confirmed that the brown-rot fungi-dominated degradation of lignocellulosic residues resulted in a greater SOM buildup than the white-rot fungi-dominated degradation.

## Introduction

Nearly one-quarter of Earth’s land surface is covered by forests in which the decomposition of woody debris continuously recycles plant biomass; consequently, varying amounts of leachate and particulate matter enter the soil system over a period of decades^1–4^. Soil carbon (C) and nitrogen (N) concentrations are greatly influenced by the initial composition of litter (e.g., woody debris) when soil mineralogy and texture are similar^1,5,6^. Moreover, soil C and N concentrations can also be affected by the pathways undertaken and the complex compounds produced by the degradation of specific lignocellulosic residues^7–9^, as well as the soil microbes that are present or active in the working systems^10,11^.

Wood residues are primarily composed of lignin, cellulose and hemicellulose^12,13^, and wood-rotting fungi dominate the decomposition process^14–16^. For instance, white-rot fungi can either degrade lignin and leave relatively high levels of white-colored cellulose or hemicellulose along with degraded lignin fragments; alternatively, these fungi can degrade lignin and cellulose simultaneously^7,14,17^. Although brown-rot fungi can promote the extensive decay of cellulose and hemicellulose through both enzymatic and non-enzymatic mechanisms, the lack of lignin-degrading extracellular phenoloxidases may lead to the accumulation of depolymerized and demethylated brown-colored lignin in litter and surface soils^15,18–21^. During the decomposition processes dominated by white- or brown-rot fungi, soluble wood decay products can be leached into soils and adsorbed onto the hydroxide surfaces of cations in the soil matrix, thereby affecting the formation and protection of soil organic matter (SOM)^1,18,22^. This process can evolve in a cascading cycle of abiotic adsorption and microbial uptake that can be described in terms of a soil dynamic continuum^23^.

The effects of the soil microbial community on the leached wood decay products determine SOM storage because distinct microbial compositions and byproducts have different effects on the formation and stabilization of organic substances. For instance, high fungal/bacterial ratios may be associated with increased C sequestration in soils^10,11^. In addition, the soil microbial necromass can underpin the formation and stabilization of long-lived SOM^24–27^, which is particularly pertinent when mineral and particle sorptive interactions favor matrix protection and the recalcitrance of certain organic products of secondary microbial synthesis^27–29^. By contrast, dissolved decay products may also have the potential to facilitate the loss of previously stable soil C through microbial excavation^30^, whereby soil microbes increase their rates of decomposition of original soil C with the introduction of an additional C source because of changes in substrate preference, activity, or community structure^31–33^ or the selective liberation of mineral- or physically-protected C^34^.

A considerable knowledge gap exists with regard to the linkage between the specific lignocellulosic residues of wood-decaying processes (e.g., white- vs. brown-rot) and proximal belowground microbially-mediated C and N cycling. The effects of surficial wood decay products on microbially-mediated SOM accumulation are difficult to assess quantitatively using most of the currently available techniques, such as culture-dependent methods, pyrosequencing-based approaches for nucleic acid analysis, phospholipid fatty acid analyses, substrate utilization capacities, and state-of-the-art metaproteomics^35–40^. However, microbially-mediated SOM formation induced by wood decay products can be estimated based on amino sugars, which are important cell wall components that can persist for several decades after the lysis of viable microbial cells^41,42^. Three amino sugars—glucosamine (GluN), galactosamine (GalN) and muramic acid (MurN)—and their ratios have been quantified and extensively used to indicate specific microbial necromass contents and community structure^43–45^. Fungal cell walls contain a polymer of N-acetylglucosamine, whereas the peptidoglycan of bacterial cell walls contains both N-acetylglucosamine and N-acetylmuramic acid^44,46,47^. Therefore, MurN is usually used to indicate bacterial residues, and GluN is generally considered a primary biomarker of both fungal and bacterial necromass^43,44,48^. Furthermore, variations in the GluN/GalN and GluN/MurN and GalN/MurN ratios may be closely correlated with different fungal or bacterial predominance^43,44,49^. Thus, long-lived amino sugars can be a proxy of microbial necromass abundance and composition, which are directly linked to SOM storage during wood decay.

**Figure 1:**
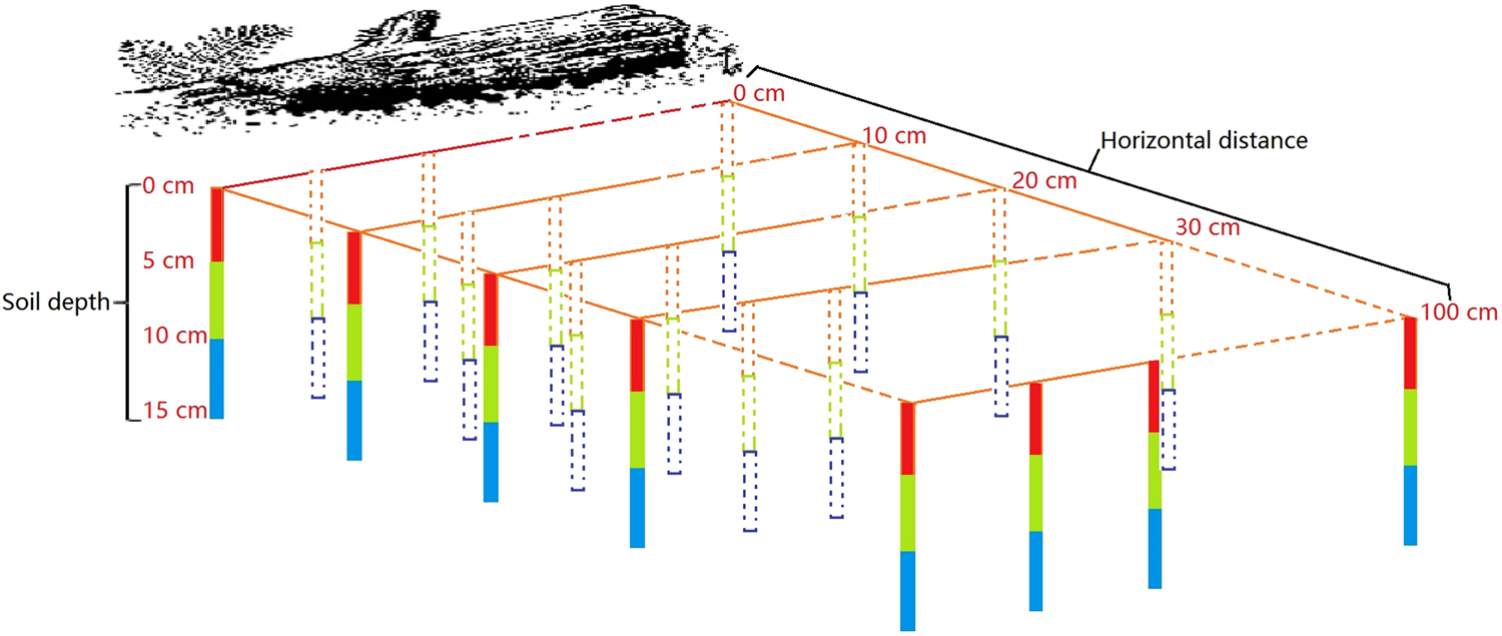
Illustration of the sampling design for soil samples around woody debris. Soil depth increments are 0–5 cm (D_0–5_, red column), 5–10 cm (D_5–10_, green column) and 10–15 cm (D_10–15_, blue column). Separate horizontal distances radiate perpendicularly from woody debris at 0 cm (i.e., directly beneath the woody debris, Zone H_0_), 10 cm (Zone H_10_), 20 cm (Zone H_20_), 30 cm (Zone H_30_) and 100 cm (Zone H_100_).

As described above, the brown- and white-rot fungal decay pathways progressively alter the lignocellulose composition of woody debris but with different chemical outcomes for both lignin constituents and structural carbohydrates. White rot degrades lignin, leaving cellulose or hemicellulose largely intact, whereas brown rot, which decomposes cellulose or hemicellulose, results in highly concentrated lignin components. The products, i.e. leachate^50^ or particulate matter^51^ resulting from these two decomposition pathways, can be expected to exert distinct effects on soil microbial metabolism, necromass accumulation and overall soil C and N dynamics proximal to long-lived decay sources. Understanding the effects of each wood-rotting type of fungus on soil nutrient cycles can provide quantitative information on C and N allocation, residence, and stocks in forest ecosystems. To address this issue, we analyzed the concentrations of C and N and the composition of amino sugars in soil immediately below (0–15 cm) and laterally adjacent to (0–1 m) logs in advanced stages of either brown- or white-rot decay (Fig. 1) in a mixed-species forest within the Changbai Mountain Nature Reserve (CBMNR) of northeastern China.

## Results and Discussion

### Spatial heterogeneity of C and N and microbial necromass caused by wood decay

**Figure 2:**
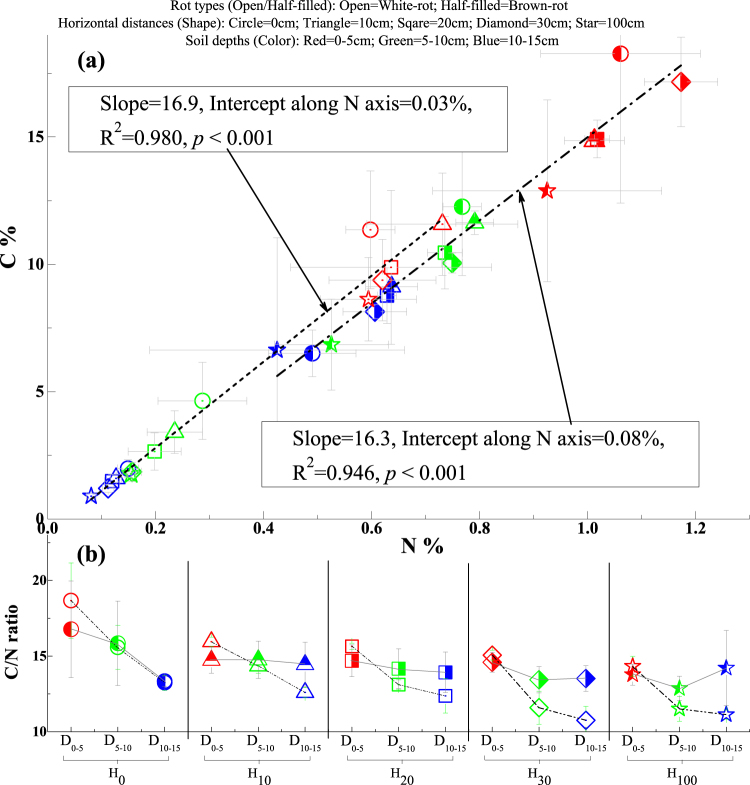
The C and N abundances and C/N ratios at three different soil depths from beneath the coarse woody debris to a perpendicular distance of 100 cm as a result of the brown- or white-rot fungi. (**a**) C and N concentrations and their relationships; (**b**) C/N ratios. Horizontal distances are separately indicated as H_0_ (0 cm), H_10_ (10 cm), H_20_ (20 cm), H_30_ (30 cm) and H_100_ (100 cm). Soil depths are represented by D_0–5_ (0–5 cm), D_5–10_ (5–10 cm) and D_10–15_ (10–15 cm).

The soil total C, total N and amino sugar concentrations increased in regions proximal to woody debris in the current study. For instance, the soil C concentrations were lower in Zone H_100_ (5.8%) than in Zone H_0_ (8.5%), and the soil N concentrations varied little from Zone H_0_ to Zone H_30_ (~0.5%) but were lower in Zone H_100_ (0.4%) (Table 1, Fig. 2a). Compared with the surface soil (D_0–5_), the subsurface soil C and N concentrations decreased by 53.2–67.7% and 50.0–62.5%, respectively, at depths of 5–10 (D_5–10_) and 10–15 cm (D_10–15_) (*p* < 0.05) (Table 1, Fig. 2a). Consistently, the fungal and bacterial necromass C concentrations were higher (ca. 50%) in the surface than in the subsurface soils (*p* < 0.05). A horizontal stratification effect was detected for fungal residues between Zones H_10_ and H_100_ (Table 1). In a previous study, the soil beneath woody debris in *Eucalyptus* woodlands in southeastern Australia contained higher C and N concentrations than soil located 80 cm horizontally from the woody debris^52^. Additionally, as a result of well-decayed woody debris, surface soils have lower pH and higher exchangeable acidity (especially those associated with brown-rot fungi, Supplementary Table S1), partly as a result of the accumulation of more polyphenols directly beneath decaying wood^53^. Such large spatial heterogeneity of soil properties resulting from wood decay can be explained by the translocation of the soluble fractions of degraded wood products via water percolation into the soil^1,54^. These transported components enrich C and N concentrations in SOM pools either through adsorption to soil particles or via immobilization in soil microbial community biomass^5,6,53^.

**Table 1:** Analysis of variance results of the effects of rot types, horizontal distances and soil depths on soil C and N and microbial necromass.

Variables		Rot types	Horizontal distances	Soil depths
C (%)	Rank	white-rot(4.8) < brown-rot(11.2)	H_0_(8.5) ≈ H_10_(8.1) ≈ H_20_(7.4) ≈ H_30_(7.2) ≈ H_100_(5.8)	D_0–5_(12.4) > D_5–10_(5.8) ≈ D_10–15_(4.0)
	H-value	(25.3)^***^	(2.7)	(33.6)^***^
N (%)	Rank	white-rot(0.3) < brown-rot(0.8)	H_0_(0.5) ≈ H_10_(0.5) ≈ H_20_(0.5) ≈ H_30_(0.5) ≈ H_100_(0.4)	D_0–5_(0.8) > D_5–10_(0.4) ≈ D_10–15_(0.3)
	H-value	(30.7)^***^	(1.8)	(31.5)^***^
C/N	Rank	white-rot(13.7) ≈ brown-rot(14.3)	H_0_(15.6) ≈ H_10_(14.4) ≈ H_20_(13.9) ≈ H_30_(13.0) ≈ H_100_(12.8); H_0_ > H_30_^a^	D_0–5_(15.5) > D_5–10_(13.6) ≈ D_10–15_(12.8)
	H-value	(1.6)	(10.1)^*^	(22.8)^***^
Fun C (mg g^−1^)	Rank	white-rot(19.7) < brown-rot(52.7)	H_0_(35.1) ≈ H_10_(35.1) ≈ H_20_(34.6) ≈ H_30_(33.1) ≈ H_100_(27.3)	D_0–5_(52.9) > D_5–10_(28.5) ≈ D_10–15_(18.9)
	H-value	(34.2)^***^	(1.8)	(24.8)^***^
Bac C (mg g^−1^)	Rank	white-rot(3.7) < brown-rot(10.0)	H_0_(6.6) ≈ H_10_(5.8) ≈ H_20_(6.0) ≈ H_30_(7.4) ≈ H_100_(5.5)	D_0–5_(9.2) > D_5–10_(5.4) ≈ D_10–15_(4.4)
	H-value	(32.0)^***^	(1.3)	(18.6)^***^
Fun/Bac C	Rank	white-rot(5.3) ≈ brown-rot(6.2)	H_0_(5.7) ≈ H_10_(6.1) ≈ H_20_(6.1) ≈ H_30_(5.2) ≈ H_100_(5.4)	D_0–5_(6.8) ≈ D_5–10_(5.8) ≈ D_10–15_(4.6); D_0–5_ > D_10–15_
	H-value	(2.1)	(3.4)	(6.3)^*^

Note: Mean values of specific treatment are presented in parentheses. Variables include soil carbon (C) and nitrogen (N), fungal necromass carbon (Fun C) and bacterial necromass carbon (Bac C). White- and brown-rot fungi are shortened to white- and brown-rot, respectively. Horizontal distances are indicated as H_0_ (0 cm), H_10_ (10 cm), H_20_ (20 cm), H_30_ (30 cm) and H_100_ (100 cm). Soil depths are indicated as D_0–5_ (0–5 cm); D_5–10_ (5–10 cm) and D_10-15_ (10–15 cm). Differences between factor levels were detected using Kruskal-Wallis test. ^***^ *p* < 0.001; ^**^ *p* < 0.01^; *^ *p* < 0.05; ^a^ *p* < 0.1.

The soil C/N ratios were higher at D_0–5_ (15.5) than at D_5–10_ (13.6) and D_10–15_ (12.8) (*p* < 0.05) and decreased from Zone H_0_ (15.6) to Zone H_100_ (12.8) (*p* < 0.1) (Table 1, Fig. 2b). These C/N ratios are much lower than those in rotten wood^51,55,56^. Similar relationships have been demonstrated for a Douglas-fir forest stand^57^ in which the C/N ratios of mineral soil below woody debris were lower than those of any aboveground detrital component and decreased from 26 to 18 along the soil profile. The decline in the soil C/N ratio with increasing horizontal distance from woody debris is consistent with the results presented by Goldin and Hutchinson^52^, who found that soil C/N ratios were, on average, 15.1 at 0 cm and 13.1 at 80 cm from woody debris. Such plant-derived molecular imprints on soil C/N ratios—being much lower than and increasing closer to aboveground woody debris—are unequivocally a consequence of both the marked increase in N concentration during the process of litter decay and the highly concentrated C resources in woody debris^6,58,59^. Increasing concentrations of N during wood-decaying processes are explained by N immobilization by directly contacted fungal hyphae and the interactions with diazotrophs^57,60,61^. Simultaneously, highly enriched recalcitrant wood C compounds probably result in a constant C fraction in wood decay leachates^59,62^.

### Greater SOM buildup beneath the brown- compared with the white-rot sets

The concentrations of C, N and microbial necromass proxies were higher under the brown-rot decayed wood than under the white-rot decayed wood (Table 1, Supplementary Tables S2 and S3): (1) the C and N concentrations were approximately 1.3- and 1.7-fold higher, respectively (*p* < 0.05), and (2) the fungal and bacterial necromass C concentrations were approximately 1.7-fold higher (*p* < 0.05). Brown-rot woody debris contains high concentrations of soluble aromatic and phenolic compounds and metals^21^; its presence may thus promote chemical and physical protection of both itself and the existing SOM from degradation, consequently increasing the mean residence time of soil C and contributing to further SOM “stabilization” along a soil profile or at discrete distances from the woody debris^1,8,15,18^. By contrast, white-rot fungi have aggressive enzyme systems for both hydrolytic and ligninolytic activities, which allow the complete decay and disruption of the structural and chemical components of cell walls^17,63,64^. The soluble sugars and other rapidly metabolized components progressively released at low rates from white-rot woody debris are most likely available to nearby R-strategists, which promote the decomposition of extant SOM to acquire available N, leading to increased mineralization of the SOM pools^65–67^. The negative relationships between the bacterial residue index (MurN) and lignin monomers V, S, and C (Table 2) might indicate a reduction in the bacterial growth caused by the acidic products released during the white-rot decay process. Such inhibition of bacterial growth induced by white-rot decay products was significantly reduced as lignin oxidation proceeded because lignin fragments Ac/Al (V) and C/V ratios, which increase with lignin oxidation and soil depth^68^, were positively correlated with bacterial residue concentrations in this study (Table 2).

**Table 2:** Correlation between lignin monomers and amino sugars in white-rot (brown-rot) sets.

Amino sugars Lignin monomers	GluN (mg g^−1^C)	GalN (mg g^−1^ C)	MurN (mg g^−1^ C)	Total amino sugars (mg g^−1^ C)
V (mg g^−1^ C)	−0.47 (−0.36)	−0.47 (−0.43)	−**0.64** (−0.36)	−0.48 (−0.46)
S (mg g^−1^ C)	−0.43 (0.34)	−0.36 (0.03)	−**0.61** (−0.13)	−0.43 (0.24)
C (mg g^−1^ C)	−0.41 (0.21)	−0.41 (−0.31)	−**0.59** (−0.34)	−0.43 (−0.01)
S + V + C (mg g^−1^ C)	−**0.53** (−0.23)	−**0.52** (−0.41)	−**0.73** (−0.38)	−**0.54** (−0.36)
Ac/Al (V)	0.25 (−0.41)	0.19 (−0.20)	0.40 (0.09)	0.24 (−0.36)
Ac/Al(S)	−0.15 (0.09)	−0.11 (0.06)	−0.22 (−0.19)	−0.15 (0.07)
S/V	0.05 (0.16)	0.07 (0.50)	0.10 (0.45)	0.06 (0.36)
C/V	**0.54** (0.32)	**0.51** (0.33)	**0.63** (0.39)	**0.54** (0.39)

Note: The vanillyl (V), syringyl (S) and cinnamyl (C) phenolic units separated using the cupric oxide (CuO) method were used as a measure of the lignin monomer concentrations in soil. The oxidation state of V or S lignin fragments is indicated as Ac/Al (i.e., the acid/aldehyde ratio). S/V indicates the syringyl/vanillyl ratio, and C/V indicates the cinnamyl/vanillyl ratio. Three amino sugars, namely glucosamine (GluN), galactosamine (GalN) and muramic acid (MurN), were quantified to reveal the soil microbial necromass composition. The values in the table are “r” values, i.e., the correlation between lignin monomers and amino sugars in the white-rot (brown-rot) sets. Significant values (*p* < 0.05) are indicated in boldface.

Moreover, brown-rot fungal growth, which selectively enriches lignin polymers that are bound to over 50% of wood N^69^, most likely favors N condensation, whereas delignification with simultaneous N utilization by white-rot fungal growth is linked to low N concentration and availability in decayed woody debris^70–72^. Furthermore, brown-rot woody residues are more likely to accumulate N compared with white-rot residues because brown-rot fungi can efficiently accelerate N_2_ fixation^61,73^ and are therefore a more favorable ecological niche for mycorrhizal growth^74^. These different mechanisms most likely resulted in the 0.05% higher N concentration in the brown- than in the white-rot-associated soil, which was evident in the comparison of the intercepts of the regression curves along the N% axis (i.e., if the C concentration equaled 0, the N concentration should be 0.08% for the brown-rot sets and 0.03% for the white-rot sets) (Fig. 2a). The values of these intercepts along the N% axis on average accounted for ca. 10% of the N concentrations in the brown- and white-rot-associated soils, as the mean N concentrations in these soils were 0.8% and 0.3% (Table 1), respectively.

### Linkage of microbial necromass to C and N concentrations in soil

**Figure 3:**
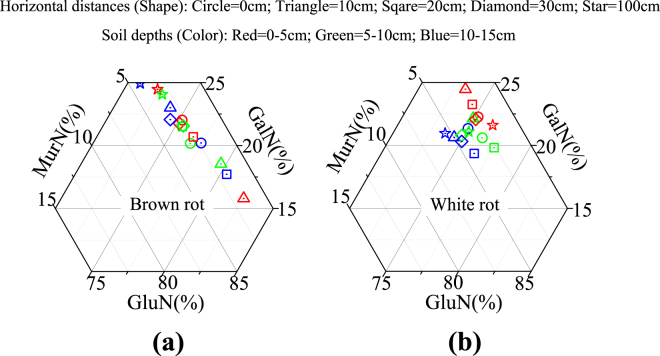
The ternary diagram of amino sugars at three different soil depths from beneath the coarse woody debris to a perpendicular distance of 100 cm. (**a**) The ternary diagram of amino sugar compositions associated with brown rot; (**b**) The ternary diagram of amino sugar compositions associated with white rot.

Using the ternary diagram of the relative abundances of three amino sugars, we classified the microbial necromass composition into two contrasting patterns between the brown- and white-rot-associated soils (Fig. 3). With respect to the soil amino sugar composition in the brown-rot sets (Fig. 3a), Zone H_100_ displayed the highest GalN% but the lowest GluN% (i.e., primary fungal residue index). This was significantly distinct from Zones H_0_ to H_30_, in which the opposite trend was observed (*p* < 0.05, except for D_10–15_ in Zone H_10_). By contrast, the ternary plot of amino sugar composition in the white-rot affected soils clearly revealed a significant shift in the MurN (bacterial residue index) proportion with soil depth (Fig. 3b), i.e., D_0–5_ < D_5–10_ < D_10–15_ (*p* < 0.05).

Acidic leachates from woody debris significantly decreased the pH of nearby surface mineral soils^53^. Jiao *et al*.^75^ reported that the pH values (soil: water = 1: 2.5) of the same rot-associated soils as in this study varied from 3.8 to 5.5 in the brown-rot sets and were lower than those in the white-rot sets (5.0–5.7) (Supplementary Table S1). Brown-rot fungi produce more oxalic acid and lignin phenolics, which result in lower pH and inhibit the activities of bacteria in brown- compared with white-rot systems^65,76,77^. Additionally, white-rot fungi oxidize acidic products such as cellobionic acid and oxalic acid^76,78^. These findings support (1) the significant correlations between the bacterial residue index (MurN) and lignin monomers and their ratios (Table 2, and (2) higher relative abuandances of bacteria in the white- than brown-rot sets (Fig. 3). Fungal/bacterial ratios are positively correlated with the soil C/N ratios because bacteria require more N per unit of biomass C assimilation than fungi^11^, and higher C assimilation efficiencies are correlated with larger proportions of fungi but not with larger proportions of bacteria^79–82^. We observed that the C/N ratios decreased to a greater extent along soil profiles with increasing horizontal distance for the white- than for the brown-rot woody debris (Fig. 2b).

The fungal decomposer composition can change during plant residue degradation due to the changing quality of available decayed products^83^. Over horizontal distances that radiate perpendicularly from woody debris, the translocated soluble wood products may present a gradient of substrate quality, i.e., soil closer to well-decayed woody debris has a higher concentration of recalcitrant substrates. Because the wood decay products exist longer in the brown- than in the white-rot systems, the spatial allocation of wood decay products should be more likely to occur in brown-rot systems. Consequently, it is reasonable to observe a fungal composition shift along horizontal distances in the brown-rot associated soils (Fig. 3a).

**Figure 4:**
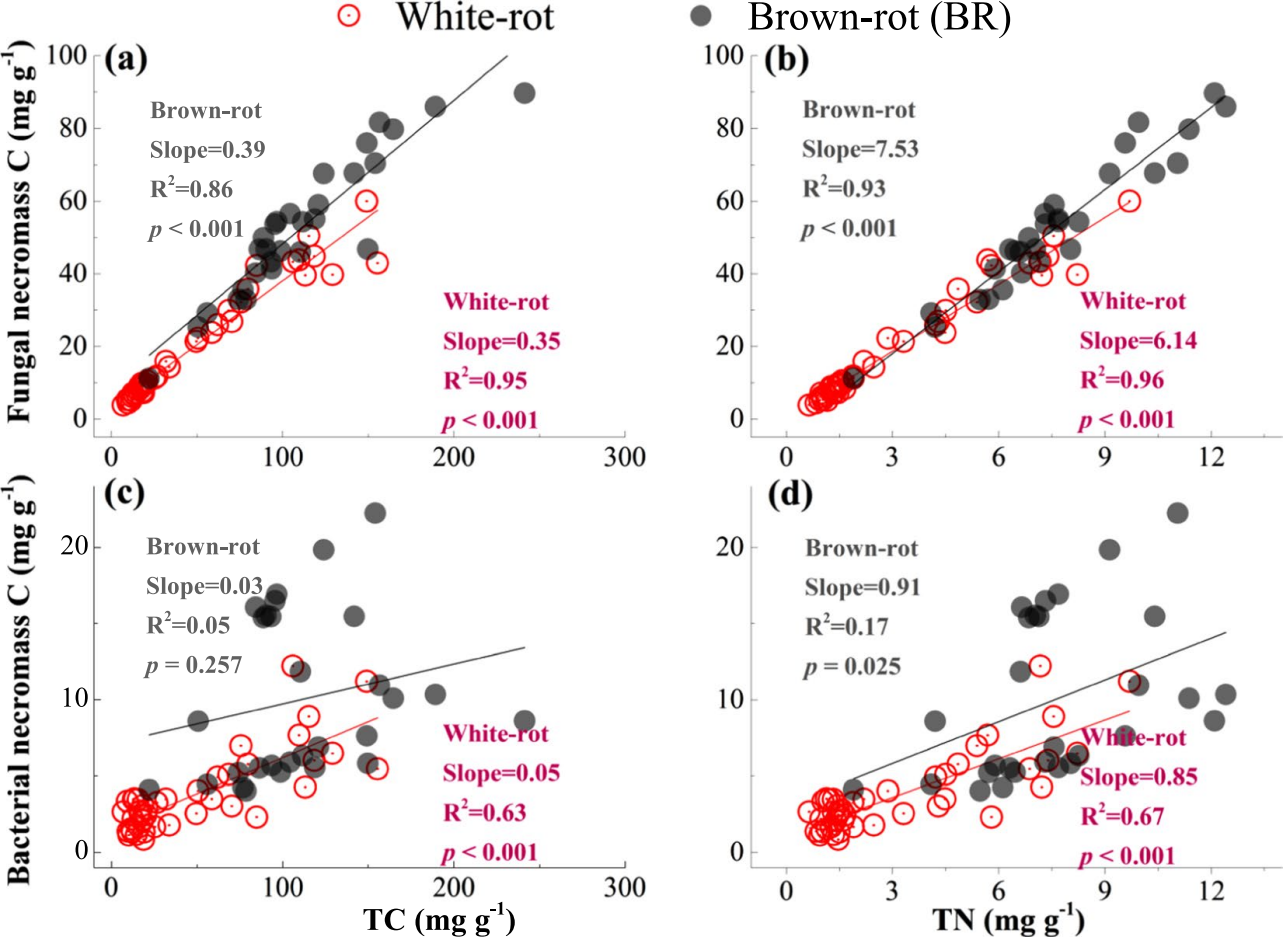
Correlations between microbial necromass and C and N concentrations at three different soil depths from beneath the coarse woody debris to a perpendicular distance of 100 cm. (**a**) Correlations between fungal residue C and C concentrations; (**b**) correlations between fungal residue C and N concentrations; (**c**) correlations between bacterial residue C and C concentrations; (**d**) correlations between bacterial residue C and N concentrations.

The slopes derived from the linear regressions of fungal necromass vs. C and N were greater in the brown- than in the white-rot sets (Fig. 4a and b). By contrast, the bacterial residues were not as well correlated with the soil C and N in the brown-rot-associated soils as they were in the white-rot-associated soils (Fig. 4c and d). These findings suggested that the bacterial residues in the brown-rot-associated soils were unresponsive in proportion to the increased fungal necromass C concentrations and therefore showed a lack of correlation with C and N concentrations, as observed for the white-rot-associated soils. The combination of higher C and N concentrations and a higher fraction of fungi in the brown- than in the white-rot systems (Table 1, Fig. 3) suggested that a microbial community with higher proportions of fungi under brown-rot woody debris preferentially maintained larger SOM stocks than the soil associated with white-rot woody debris in which bacterial residues were concentrated.

## Materials and Methods

### Description of the research area

The study site is in a mixed spruce, fir, birch, oak and aspen forest within the CBMNR (42°23′N, 128°05′E, 802 m a.s.l.). *Picea jezoensis* and *Abies nephrolepis* are the dominant tree species. Precipitation occurs primarily from June to September and averages 700 mm annually; the annual average temperature is 2.8 °C^84^. The soil at the sampling site is an andosol that developed from volcanic ash^85^.

### Identification of decayed logs

We chose decayed logs (ca. 20 cm in diameter) that were in contact with soil and in the final decay stage^62^, which could be identified separately as decayed by brown-rot or white-rot fungi based on their fragmentation morphology^2,16,55^. The brown-rot woody debris was characteristically “brittle and broke easily into cube-like pieces”, whereas the white-rot woody debris was “spongy with longitudinal fragmentation”^63^ (see the pictures of woody debris in Supplementary Figure S4). The dominant fungal species responsible for the specific brown and white rot were identified based on their fruiting bodies. The white-rot-degraded logs of *Abies nephrolepis* were decomposed by the basidiomycete *Antrodiella gypsea* (Yasuda) T. Hatt. & Ryvarden, which is a common fungus found primarily on gymnosperm wood that has a preference for *Abies* ^86^. The brown-rot-decayed logs of *Abies nephrolepis* were decomposed by *Fomitopsis pinicola* (Sw.) P. Karst, which is one of the most common wood decay basidiomycete fungi and is frequently found on both gymnosperms and angiosperms^86,87^. The vanillyl (V), syringyl (S) and cinnamyl (C) phenolic units separated using the cupric oxide method^88^ were used as measures of the concentrations of lignin monomers in the brown- and white-rot-associated soils. The results showed that much higher lignin concentrations occurred under the brown- than under the white-rot-associated soils (Supplementary Figure S5). For instance, at horizontal distances of 20 and 30 cm, the lignin concentrations were 54% and 72% greater, respectively, in the brown-rot-associated soils than in the white-rot-associated soils; at a depth of 5–10 cm, the lignin concentrations were on average 318% higher under the brown- than under the white-rot sets. These findings are consistent with the fact that lignin is highly degraded and removed in white-rot sets, whereas brown-rot sets are characterized by the accumulation of depolymerized and demethylated lignin.

### Sampling and processing of soil

Using a 2.5-cm-diameter auger, soil samples were collected on 3 September 2011 at three soil depths in five parallel zones that radiated perpendicularly from the chosen woody debris (Fig. 1). Soil was collected at depths of 0–5 (D_0–5_, red column), 5–10 (D_5–10_, green column) and 10–15 cm (D_10–15_, blue column), and the horizontal distances from decomposing logs were 0 (i.e., directly beneath the woody debris, Zone H_0_), 10 (Zone H_10_), 20 (Zone H_20_), 30 (Zone H_30_) and 100 cm (Zone H_100_). At least four soil cores were collected from each sampling zone. The width of each zone was less than 2.5 cm (diameter of the soil auger). Soil samples were collected on both sides of three logs for white rot and of two logs for brown rot, and soils were immediately homogenized thoroughly while moist (i.e., 5 rot sets × 5 horizontal zones × 3 depths = 75 composite samples). The soil samples were sieved through a 2-mm mesh to remove visible roots and rocks and were then air-dried and milled (<250 μm) to determine C, N, lignin and amino sugar concentrations.

### Quantification of soil C, N, amino sugars and microbial necromass

Soil C and N concentrations were determined by dry combustion using an elemental analyzer (vario Macro cube; Elementar Analysensysteme GmbH, Hanau, Germany), and the C/N ratios are presented as mass ratios. Soil amino sugars, including GluN, GalN and MurN, were purified, derivatized and quantified according to Zhang and Amelung^89^ with modifications by Liang *et al*.^90^ In brief, the soil samples were digested by acid hydrolysis followed by iron and salt removal via precipitation and subsequent derivatization to aldononitrile derivatives. Gas chromatography separation and detection of derivatized amino sugars were performed using an HP-5 silica column (25 m × 0.32 mm × 0.25  μm) and flame ionization detector connected to an Agilent 6890 A GC (Agilent Tech. Co., USA).

Proxies for the relative necromass abundances of bacteria and fungi were determined using relative proportions of the extracted amino sugars^91,92^. MurN primarily occurs in bacteria^47,48^, whereas both fungal and bacterial cell walls contain GluN^44,46^; a MurN:GluN ratio of 1:1 is the proportion found in bacterial cell walls^91,93^. The microbial necromass C sources, i.e., fungal and bacterial residue C concentrations, were calculated as follows: fungal C mg g^−1^ dry weight = 9 × 179.2 × (mol GluN − mol MurN), where 179.2 is the molecular weight of GluN and 9 is the conversion value of fungal GluN to fungal C; bacterial C mg g^−1^ dry weight = 45 × MurN C, where 45 is the conversion index from MurN C to bacterial residue C^91,92^.

### Statistical analyses

Although this study only included two replicates for brown rot and three replicates for white rot, it was important to acquire predictions of soil organic C buildup and microbial necromass composition induced by specific rot decay (i.e., the brown vs. white rot) at our study site. Statistical analyses were performed (including 2 × brown-rot and 3 × white-rot sets) using Statistica 10 software (StatSoft, Inc., USA). The effects of soil depth, horizontal distance from decayed logs, and wood-rotting types of fungi were detected using a nonparametric test (the Kruskal-Wallis test) as the distribution of replicates is not homogenous between wood-rotting types. One-way analysis of variances (ANOVAs) were performed for each variable, followed by multiple comparisons between group levels (Kruskal-Wallis test, *p* < 0.05). Regression analysis and figure construction were performed using Origin 8.5 (OriginLab Corp., USA). A χ^2^ test was used to assess the statistical significance between different subsamples in a ternary graph^94^.

### Data availability

The authors declare that the data in the current manuscript are available upon request.

## Conclusions

Fungal wood decay resulted in high spatial heterogeneity of soil C and N and microbial necromass concentrations. The soil C and N concentrations of the brown-rot sets were higher than those of the white-rot sets, which suggested that brown rot contributed more to SOM buildup than white rot. A microbial community process, rather than a single-species dynamic, mediates plant-derived molecular contributions to SOM buildup. Because different microbial groups can form both unique and long-standing necromass components, amino sugar composition analysis can identify specific soil microbial footprints (i.e., the legacy of the microbial community) and provide a solid foundation to explain the microbial controls of and interactions within the soil nutrients stored during the process of fungal wood decay. Our findings of strong correlations between the fungal necromass and soil C and N concentrations in the brown-rot sets confirmed that higher proportions of fungi rather than bacteria contributed to a greater buildup of SOM. In future studies, which include increasing organic matter incorporation into soil fractions and mitigating CO_2_ release in terrestrial ecosystems, the focus should be on the mechanisms of SOM stabilization and the genesis driven by specific types of wood decay (particularly for brown rot) in forests, in addition to the important contribution of microbial necromass in microbially-mediated soil processes.

## Electronic supplementary material

Supplementary information
