# Supplementary material for: Spatial Heterogeneity of SOM Concentrations Associated with White-rot Versus Brown-rot Wood Decay
Source: Sci Rep. 2017 Oct 23;7:13758. doi: 10.1038/s41598-017-14181-7 (PMC5653805; doi:10.1038/s41598-017-14181-7)
Supplement: Supplementary file 1 — Supplementary information [file 41598_2017_14181_MOESM1_ESM.pdf]

Title page

Spatial Heterogeneity of SOM Concentrations Associated with White-rot Versus Brown-rot Wood Decay

Zhen Bai, Qiang Ma*, Yucheng Dai, Haisheng Yuan, Ji Ye, Wantai Yu*

Institute of Applied Ecology, Chinese Academy of Sciences, Shenyang, 110016, China. Correspondence and requests for materials should be addressed to Wantai Yu (Email: wtyu@iae.ac.cn) and Qiang Ma (Email: qma@iae.ac.cn).

Table S1 Soil pH values in different treatments

Soil depths \ Distances	0cm	10cm	20cm	30cm	100cm
0~5cm	5.00（3.76）	5.50（4.25）	5.12（4.26）	5.30（4.84）	5.65（5.47）
5~10cm	5.05（4.12）	5.23（4.36）	5.14（4.89）	5.29（5.25）	5.17（5.23）
10~15cm	5.00（4.24）	5.08（4.40）	5.18（5.14）	5.23（5.28）	5.18（4.82）

Note: The pH values (soil : water = 1 : 2.5) were separately indicated as the means of white-rot (brown-rot), which cited from: Jiao *et al*. Different progress of log decay on forest soil organic carbon and total nitrogen in Changbai Mountain. *Chin. J. Soil Sci.* **46**, 1096–1102 (in Chinese) (2015).

Table S2 Soil C and N concentrations in different treatments

Soil depths \ Distances		0cm	10cm	20cm	30cm	100cm
C%	0~5cm	11.35（18.27）	11.57（14.86）	10.57（14.92）	9.38（17.16）	8.63（12.89）
	5~10cm	4.64（12.26）	3.41（11.63）	2.27（10.46）	1.85（10.05）	1.77（6.85）
	10~15cm	1.99（6.51）	1.60（9.12）	1.49（8.77）	1.21（8.14）	0.90（6.63）
N%	0~5cm	0.60（1.06）	0.73（1.01）	0.67（1.02）	0.62（1.17）	0.59（0.93）
	5~10cm	0.29（0.77）	0.24（0.79）	0.17（0.74）	0.16（0.75）	0.15（0.53）
	10~15cm	0.15（0.49）	0.13（0.64）	0.12（0.63）	0.11（0.61）	0.08（0.43）

Note: The values are separately indicated as the means of white-rot (brown-rot).

Table S3 Soil fungal and bacterial necromass C in different treatments

Soil depths \ Distances		0cm	10cm	20cm	30cm	100cm
Fun-C(mg/g)	0~5cm	39.69（78.70）	46.93（75.97）	34.85（74.76）	36.33（78.23）	38.88（61.55）
	5~10cm	19.48（50.25）	15.94（56.73）	12.15（50.97）	9.17（55.53）	8.68（36.04）
	10~15cm	9.86（31.07）	7.34（45.68）	7.34（40.96）	6.30（36.55）	4.58（28.62）
Bac-C (mg/g)	0~5cm	6.71（14.24）	7.68（7.63）	5.80（13.21）	7.15（16.30）	4.92（12.79）
	5~10cm	3.38（11.16）	2.96（6.61）	1.57（10.54）	2.63（11.41）	2.22（7.07）
	10~15cm	2.53（4.85）	2.62（10.53）	2.08（4.76）	1.97（10.05）	1.74（7.98）

Note: The values are separately indicated as the means of white-rot (brown-rot). Fun-C indicates the fungal necromass C concentrations; Bac-C indicates the bacterial necromass C concentrations.

Figure S4

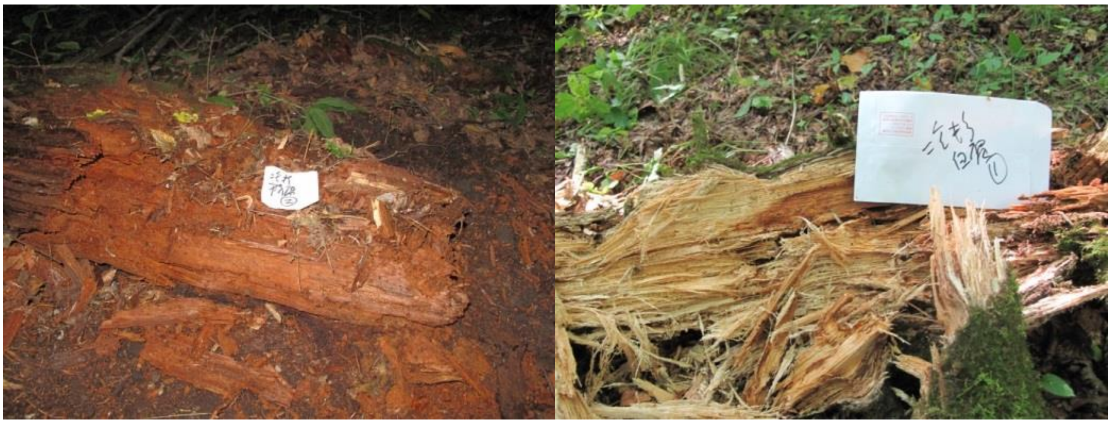

Figure S4 The brown- (left) and white-rot (right) coarse woody debris (photo by Zhen BAI).

Figure S5

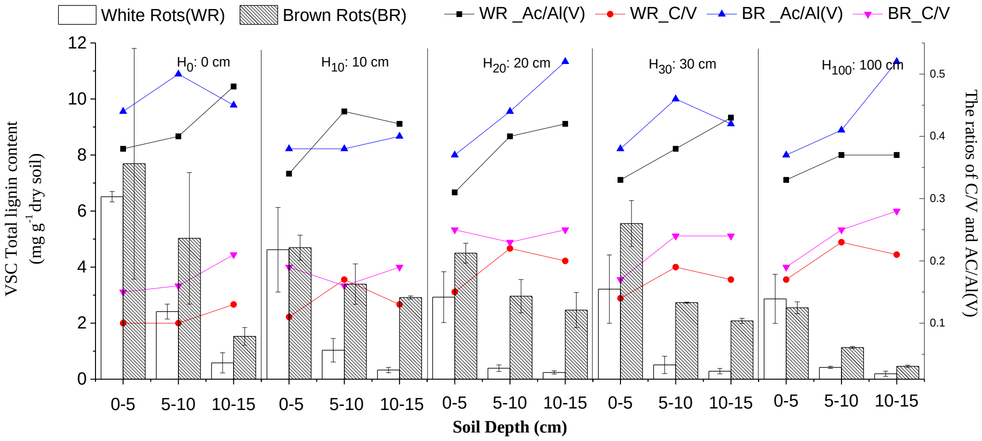

Figure S5 The lignin contents in the brown- and white-rot associated soils. The oxidation state of V lignin fragments is indicated as Ac/Al(V) (i.e. the ratios of acid and aldehyde monomers within the V lignin fragment). The C/V indicates the ratios of cinnamyl and vanillyl. Soil depth increments are 0-5 cm, 5-10 cm and 10-15 cm; horizontal distances perpendicularly radiate away from woody debris separately at 0 cm (i.e. immediately underneath the woody debris, Zone H_0), 10 cm (Zone H_{10}), 20 cm (Zone H_{20}), 30 cm (Zone H_{30}) and 100 cm (Zone H_{100}).
